# The effects of sleep and targeted memory reactivation on the consolidation of relevant and irrelevant information

**DOI:** 10.3389/frsle.2023.1187170

**Published:** 2023-05-25

**Authors:** Christine Barner, Ann-Sophie Werner, Sandra Schörk, Jan Born, Susanne Diekelmann

**Affiliations:** ^1^Medical Faculty, Institute of Medical Psychology and Behavioral Neurobiology, University of Tübingen, Tübingen, Germany; ^2^Werner Reichardt Centre for Integrative Neuroscience, University of Tübingen, Tübingen, Germany; ^3^Department of Psychiatry and Psychotherapy, University Hospital Tübingen, Tübingen, Germany

**Keywords:** memory consolidation, sleep, targeted memory reactivation (TMR), future relevance, selectivity

## Abstract

**Introduction:**

Sleep is assumed to facilitate the consolidation of new memories in an active process of covert reactivation of the underlying memory representations. Recent evidence suggests that this process is selective by favoring memories that are of future relevance, and can be externally triggered by learning-associated sensory cues presented during sleep [i.e., targeted memory reactivation (TMR)]. In the present study, we (1) set out to confirm the preferential sleep effect for relevant information, and then asked whether (2) simultaneous TMR of relevant and irrelevant information facilitates the advantage for relevant information, and (3) whether the preferential benefit of sleep and TMR for relevant information persists over time.

**Methods:**

To test these questions, participants explicitly learned two sets of picture-location associations, of which one set was instructed (after encoding) to be relevant and the other to be irrelevant for later testing. In Experiment 1, memory was tested after ~12 h of night sleep (*n* = 28) or daytime wakefulness (*n* = 28) as well as again after ~1 week.

**Results:**

Results showed overall better memory retention after sleep compared to wakefulness after 12 h as well as after 1 week. The relevant memories were overall retained better than the irrelevant memories. Interestingly, a trend toward a stronger sleep benefit for the relevant memories emerged after 1 week, although this effect failed to reach significance. In Experiment 2, learning of the relevant and irrelevant picture-location associations took place in the presence of an odor. During subsequent sleep, in the first phase of slow wave sleep (SWS), participants were either presented with the odor again (*n* = 23) or received an odorless vehicle (*n* = 20). Memory retention was assessed after the first SWS period (following awakening) as well as after ~one week. As in Experiment 1, relevant memories were overall retained better than irrelevant memories. However, TMR did not differentially affect the retention of relevant and irrelevant memories.

**Discussion:**

These findings provide tentative evidence that the selective benefit of sleep for relevant memories evolves over time but is not further facilitated by TMR.

## 1. Introduction

Sleep is essential for memory consolidation, i.e., the stabilization and reorganization of representations for the long-term (Diekelmann and Born, 2010; Rasch and Born, 2013; Stickgold and Walker, 2013). According to the active systems consolidation theory, memory consolidation during sleep is a selective process, stabilizing and strengthening mainly those memories that are in any way relevant or important for the individual. Selective memory consolidation is assumed to be essential for the efficient use of limited capacities for consolidation during sleep (Feld et al., 2016). A number of studies has shown that different features can determine whether or not a memory is relevant and thus preferentially consolidated during sleep. For instance, sleep has a stronger benefit for memories when subjects expect to be tested again after sleep (Wilhelm et al., 2011; Van Dongen et al., 2012b), when they expect to be rewarded for good performance (Fischer and Born, 2009), when memory is emotionally toned (Hu et al., 2006; Payne and Kensinger, 2010; Payne et al., 2012; Lipinska et al., 2019), and when subjects form intentions for actions in the future (Scullin et al., 2011; Diekelmann et al., 2013a; Leong et al., 2019). However, there are other studies showing weaker or even no preferential consolidation of relevant memories during sleep (Brokaw et al., 2016; Wamsley et al., 2016; Barner et al., 2018; Lipinska et al., 2019; Schäfer et al., 2020), leaving open the question whether and under which conditions sleep preferentially consolidates memories that are relevant. The first aim of the present study was, therefore, to test whether sleep preferentially consolidates relevant over irrelevant memories. Because the strongest evidence for a selective sleep benefit comes from studies with the manipulation of expectancy of a retrieval test and expectancy of a reward for good performance at retrieval (Fischer and Born, 2009; Wilhelm et al., 2011; Van Dongen et al., 2012b), we combined these two features in the present study. We hypothesized that sleep facilitates the consolidation of relevant memories, for which the retrieval was expected and a reward was expected, to a stronger degree than irrelevant memories, for which the retrieval and the reward were not expected.

With regard to potential neurophysiological mechanisms, the active systems consolidation theory (Diekelmann and Born, 2010; Rasch and Born, 2013) assumes that memory consolidation during sleep relies on the covert reactivation of new memory traces during subsequent sleep, mainly during slow wave sleep (SWS; Ji and Wilson, 2007). In a hippocampal–neocortical dialogue, memory representations in the hippocampus become reactivated together with representations in cortico-cortical networks for neocortical long-term storage (Klinzing et al., 2019). There is some evidence showing that memories that are relevant to an individual become reactivated to a stronger degree during post-learning sleep. For instance, hippocampal-striatal reactivation occurred specifically in a subset of cell assemblies that had been active during encoding of rewarded information in rats (Lansink et al., 2008, 2009). Likewise in humans, using an fMRI brain decoding approach, rewarded information was found to be spontaneously reactivated to a stronger degree during post-learning SWS than non-rewarded information (Sterpenich et al., 2021).

While such memory reactivations occur mostly spontaneously during sleep, they can also be boosted by external learning-associated stimuli like odors and sounds, a technique called targeted memory reactivation (Oudiette and Paller, 2013; Hu et al., 2015, 2020; Schouten et al., 2017; Cellini and Capuozzo, 2018; Klinzing and Diekelmann, 2019; Lewis and Bendor, 2019; TMR). So far, it is unclear whether and to what degree different memories compete for reactivation during sleep. Although memory reactivation during sleep seems to be a simultaneous process of relatively large capacity, which allows the processing of several memory traces at the same time (Schechtman et al., 2021), overall reactivation capacities might be limited (Bendor and Wilson, 2012; Feld et al., 2016; Antony et al., 2018). Consequently, it is an open question whether relevant and irrelevant memories compete for the same limited resources of reactivation and whether this leads to a trade-off when relevant and irrelevant memories are reactivated at the same time. Oudiette et al. (2013) designed an elegant study, in which picture-location associations were encoded together with specific sounds and instructions for a low or high reward. Overall, the high reward picture-locations were remembered better than the low reward items, confirming preferential consolidation for relevant information. However, when some of the sounds from the low reward category were presented again during sleep, the low reward items were rescued, i.e. they were equally well consolidated than the high reward items. Thus, TMR has the potential to facilitate the consolidation of irrelevant memories when they are specifically targeted during sleep. However, in the study by Oudiette et al. (2013), only the low reward category was cued during sleep but not the high reward category. Thus, the second main goal of the present study was to test the question how well relevant and irrelevant information becomes consolidated when they are both reactivated simultaneously with TMR. Based on the assumption that memories compete for limited reactivation resources and that relevant memories are consolidated preferentially over irrelevant memories, we hypothesized that the advantage of relevant over irrelevant memories becomes even stronger when both memories are reactivated simultaneously.

Finally, we aimed at testing how the effects of sleep and TMR on relevant and irrelevant memories evolve over time. Previous studies showed preferential consolidation of relevant memories during sleep after relatively short retention intervals of 9–14 h (Fischer and Born, 2009; Wilhelm et al., 2011; Van Dongen et al., 2012b; Baran et al., 2013) but it is unclear whether the benefit of relevant information decreases, increases or persists over time. TMR effects have been robustly found after retention intervals ranging from shorter periods including 40–120 min of sleep (Diekelmann et al., 2011; van Dongen et al., 2012a; Cairney et al., 2014; Batterink and Paller, 2017; Oyarzún et al., 2017; Klinzing et al., 2018; Schechtman et al., 2020, 2021) to whole nights of sleep (Rasch et al., 2007; Fuentemilla et al., 2013; Sterpenich et al., 2014; Cairney et al., 2017; Simon et al., 2018; Joensen et al., 2022). While some studies still observed effects of TMR for retention intervals of 1 week (Hu et al., 2015; Groch et al., 2017b; Johnson et al., 2018; Simon et al., 2018), others did not find such long-lasting effects (Groch et al., 2017a; Humiston and Wamsley, 2019). For sleep-dependent memory consolidation in general, it is not well understood how the sleep benefits for memory evolve over time (Diekelmann, 2013; Cordi and Rasch, 2021). While some studies found that sleep benefits for memory persist after 1 week (Cousins et al., 2019) and even after years (Wagner et al., 2006; Lutz et al., 2017), others did not observe long-lasting effects (Abel et al., 2019). Thus, the third aim of the present study was to investigate how the effects of sleep and TMR on relevant and irrelevant memories evolve over longer time intervals. Based on the assumption that sleep selectively favors relevant memories, we hypothesized that the benefit of sleep as well as the additional benefit of TMR for relevant memories persists over time or becomes even stronger relative to irrelevant memories.

To examine the three main questions of this study, we conducted two experiments with the same 2D object-location memory task and similar experimental design. Experiment I tested (i) whether sleep preferentially consolidates relevant over irrelevant memories. Experiment II tested whether (ii) TMR during SWS preferentially facilitates relevant over irrelevant memories. Both experiments included a second test session after 1 week to examine (iii) whether the effects of sleep and TMR for relevant memories persist or become even stronger over time.

## 2. Methods

### 2.1. Design and procedure

#### 2.1.1. Experiment 1

**Figure 1:**
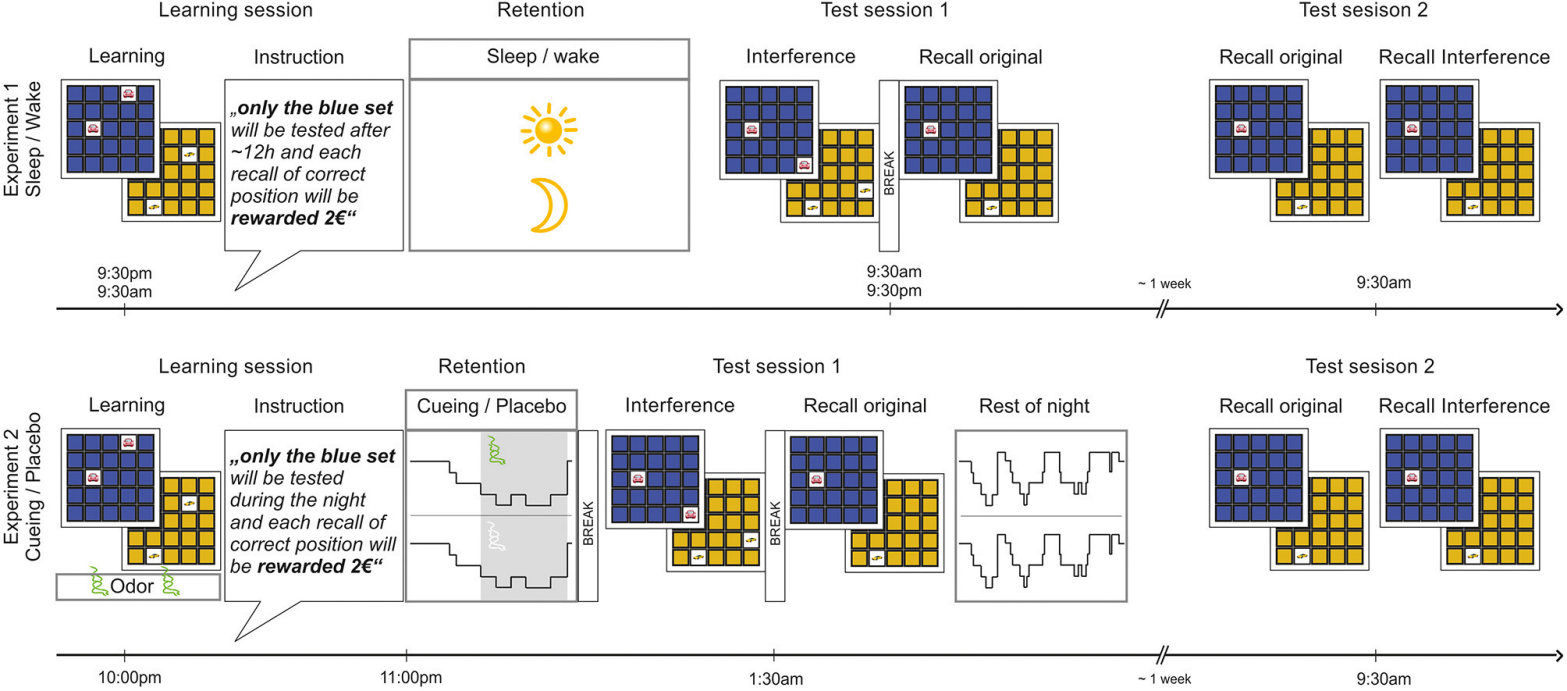
Object-location association task and designs of Experiment 1 (Sleep/Wake) and Experiment 2 (Cueing/Placebo). During the learning session, all participants encoded two sets of object-location associations (Learning) either in the morning (wake group of Experiment 1) or in the evening (all other groups). After learning, they received the relevance instruction (Instruction), outlining that only one of the two sets (counterbalanced for color) would be tested again and rewarded for correct recall. Afterwards, participants spent a retention interval (Retention) filled with either one night of sleep at home or one day of normal wakefulness (Experiment 1) or a short period of sleep in the sleep lab with cueing or placebo (Experiment 2). The first test session (Test session 1) took place after ~12 h in the next morning/evening (Experiment 1) or after awakening following the first SWS period (Experiment 2). The test session included an interference learning task (Interference) followed by cued recall of the original task (Recall original). After the first test session, participants in Experiment 1 left the lab, while participants in Experiment 2 were allowed to continue sleeping undisturbed until the next morning. After ~1 week, a second test session (Test session 2) took place for all participants in the morning, including cued recall of the original task (Recall original) and of the interference task (Recall interference). In Experiment 2, the learning session took place in the presence of an odor and the same odor (or an odorless vehicle) was presented during subsequent sleep. No odor was presented during the test sessions.

Participants were randomly assigned to Sleep (PM-AM) and Wake (AM-PM) groups, balanced for sex (Figure 1). The learning session took place in the evening between 9 PM and 10.30 PM for the sleep group and between 9 AM and 10 AM in the morning for the wake group. Participants encoded two sets of 15 object-location associations in balanced order and learning levels were assessed by immediate cued recall. Afterwards, they received the relevance instruction, i.e., they were told that one of the sets (counterbalanced independently of order) would be tested again after ~12 h and that each correct answer would be rewarded with 2 €, so that they could gain a maximum of 30 € in addition to the compensation fee. Participants were further instructed that the other set would not be tested again. They were instructed to not actively rehearse the sets and to not speak with others about the tasks.

After the learning session, sleep participants were equipped with a portable polysomnographic device for sleep monitoring at home. Additionally, subjective sleep quality was assessed with a questionnaire in the following morning. In order to monitor wake participants' activity and to ensure wakefulness throughout the day, physical activity levels were measured by an actigraphy device (Actiwatch 2; Philips Respiconics, Amsterdam, The Netherlands) attached to the subjects' non-dominant wrist. They were also asked to fill out an activity questionnaire in the evening.

The first test session (Test session 1) took place in the following morning for sleep participants and in the following evening for wake participants. First, memory stability was challenged by an interference learning task of two new sets of the same pictures at the familiar location for the first picture and a different location for the second picture of each pair. Learning level of the interference task was assessed immediately afterwards by cued recall. After a break of ~30 min, the previously announced memory test (Recall original) started between 8.30 AM and 10 AM for sleep participants and between 7.30 PM and 10 PM for wake participants. Participants were instructed that they would not only be tested for recall of the relevant set (as previously instructed), but that they would also be tested for recall of the irrelevant set. They were told that they would receive a reward of 1 € for each correct answer irrespective of which set the pictures belonged to. In the end of the experiment, a final questionnaire assessed participants' adherence to the instructions, their motivation, and to which extent they expected a reward for both object-location sets. Before leaving the lab, participants were instructed that they would complete some other tasks and questionnaires in another session 1 week later.

After on average 7.54 days (between 6 and 11 days) after the first test session, the second test session (Test session 2) took place. Immediately after awakening at home, participants were supposed to complete a questionnaire on their sleep quality during the previous night. Test session 2 started in the lab between 9 AM and 10 AM for all participants. They were reminded of the original sets differing in the color of their back sides, which they learned 1 week ago and of the two interfering sets of the same colors, which they had learned ~12 h later as a distraction from the original sets. Participants were then instructed that during the current session, recall of the original two sets would be tested first. After test completion, they were instructed that the interference sets would be tested. Both the original and the interference sets were tested by cued recall once. Another final questionnaire assessed the participants' adherence to the instructions, their motivation, and whether they expected to be tested again on the object-location sets.

#### 2.1.2. Experiment 2

Prior to the experimental night, all subjects spent an adaptation night in the sleep lab, in order to become accustomed to the sleep environment, the nasal mask and the polysomnographic setup. In the experimental night, participants were equipped with the polysomnographic electrodes together with a nasal mask for odor application upon arrival. The learning session took place at ~10 PM (Figure 1). Participants encoded two sets of 15 object-location associations in balanced order. During the learning session, they were presented with a distinct odor. The odor substance isobutyraldehyde (IBA; Sigma-Aldrich, Munich, Germany) was diluted in 10 ml odorless mineral oil (1,2 propanediol; Sigma-Aldrich, Germany) at a concentration of 1:200 and was delivered via a nasal mask to the participants. To avoid habituation, the odor was applied in a 30-s on/30-s off regimen. Apart from the odor presentation, the learning session was identical to Experiment 1. After the learning session, participants received the relevance instruction, i.e., that only one of the sets (counterbalanced independent of order) would be tested again after the first cycle of slow wave sleep (SWS) and that each correct answer would be rewarded with 2 € (as in Experiment 1).

Participants then went to bed in the sleep lab. During the first period of SWS, half of the participants (balanced for sex) were either presented with the same odor as during learning (Cueing) or they received an odorless vehicle (Placebo; 1,2 propanediol), again in a 30-s on/30-s off protocol. In total, odor was applied during 31.70 ± 12.50 epochs in the Cueing group and vehicle was applied during 39.20 ± 13.37 epochs in the Placebo group. Participants were awakened as soon as the first SWS period ended with an arousal, which was expected after around 40 min of sleep and at around 12 AM. About 30 min after awakening, to allow for recovery from sleep inertia, the first test session took place, which was identical to Experiment 1. No odors were presented during testing. As in Experiment 1, a final questionnaire assessed the participants' adherence to the instructions, their motivation, and to which extent they expected to be tested on both object-location sets.

After the first test session, participants were allowed to go back to sleep (without the nasal mask) in the lab for the rest of the night. Subjective sleep quality was assessed with a questionnaire in the following morning. Before leaving the lab, participants were instructed that they would complete some other tasks and questionnaires during another test session 1 week later (as in Experiment 1). The second test session (Test session 2) took place on average 7.23 days (between 5 and 8 days) after the first test session, starting between 9 AM and 10 AM for all participants. Before arriving in the lab, participants filled out the questionnaire on sleep quality during the previous night. The procedure of Test session 2 was identical to Experiment 1, except that participants were reminded that the interference sets were learned after the first SWS period (instead of after ~12 h in Experiment 1).

### 2.2. Participants

All participants had regular sleep-wake cycles (≥ 6 h sleep per night), no history of night shift and no shift work for at least 6 weeks prior to the experiments, participated in the study. They reported no history of any neurological, psychiatric or endocrine disorder and did not take any medication during the experiments, except for hormonal contraception and thyroid hormones. Ingestion of caffeine and alcohol was not allowed during the experimental days and participants were asked to get up before 8 AM on experimental days and to refrain from afternoon-naps and exceptional physical and mental efforts.

#### 2.2.1. Experiment 1

Overall 67 volunteers participated in Experiment 1. The final analyses included 56 subjects (58% female) between 18 and 29 years (M = 22.5, SD = 2.59). From the sleep group, seven participants had to be excluded due to the following reasons: poor sleep quality (*n* = 1), sleep latency exceeding 45 min (*n* = 2), BMI > 25 (exclusion criterion, *n* = 1), not adhering to the instruction to get up before 8 AM on experimental days (*n* = 1), errors in the experimental protocol (2x interference learning session, *n* = 1), rehearsal of the relevant but not the irrelevant object-location set (*n* = 1). From the wake group, 4 participants had to be excluded due to the following reasons: analgetic medication intake shortly before the experiment (*n* = 1), very long sleep latency in the night before the experiment (*n* = 2, sleep onset at ~2:30 AM), rehearsal of the relevant but not the irrelevant object-location set (*n* = 1). This led to *n* = 28 participants in the sleep and wake groups, respectively, for the final analysis.

#### 2.2.2. Experiment 2

Overall 69 volunteers participated in Experiment 2, of which 43 subjects (53% female) between 18 and 30 years (M = 23.89, SD = 2.72) were included in the final analysis. After the adaptation night, 7 participants were excluded due to: difficulty falling asleep (*n* = 4), allergic reaction to the electrode paste (*n* = 1), BMI > 25 (exclusion criterion, *n* = 2). After the experimental night, 19 participants were excluded due to the following reasons: sleep latencies exceeding 45 min (*n* = 7), short SWS duration (*n* = 3), subjective feeling of being unable to fall asleep (*n* = 2), technical problems with odor application (*n* = 3), error in the protocol (*n* = 1), rehearsal of the relevant object-location set (*n* = 2), dropout at Test session 2 (*n* = 1). This led to *n* = 23 participants in the Cueing group and *n* = 20 participants in the Placebo group.

### 2.3. Object-location task

For the two-dimensional object-location memory task, participants learned the positions of card pairs showing different animals and everyday objects (Rasch et al., 2007; Diekelmann et al., 2011; Klinzing et al., 2016, Figure 1). The task resembles the game “concentration”. The back sides of the cards were represented by squares and objects were presented to the participants at the respective position of the matrix. Two parallel sets were used on two 5 x 6 matrices. Each set contained 15 card pairs comprising different objects at different locations. For clear distinction of the sets, they differed in the color of their back sides (i.e. blue and yellow). During the learning session, participants encoded the two sets in balanced order, one after the other. For each set, the first card of each pair was presented alone for one second followed by the presentation of both cards for 3 s. After an inter-stimulus interval of 3 s, the next card pair was presented in the same way. The whole set of card pairs was presented twice in different orders. Immediately after these two learning rounds, recall of the spatial locations was tested using a cued recall procedure, i.e., the first card of each pair was presented and the subject had to indicate the location of the second card with a computer mouse. Depending on the correctness of choice, a green tick or a red cross was presented at the chosen location and independent of correctness, the second card was presented at the correct location for 2 s. The cued recall procedure was repeated until the subject reached a criterion of 60% correct responses. Afterwards, the second set of card pairs was learned in the same way in two consecutive cycles and to the same criterion of 60%.

The first test session (Test session 1) started with an interference learning task (Interference). The two sets of card pairs of the original learning session were presented in the same order, i.e., if the blue set was presented first during original learning, it was also the first interference set. The only difference for the interference task was that the second card of each pair was presented at a different location. The interference learning procedure was the same as the original learning procedure, including two cycles of presentation for the whole set, but cued recall was applied only once and without feedback of the correct position. After learning of the first interference set, the same procedure was applied for the second interference set. After a break of ~30 min, recall of both original object-location sets was tested in the same order as during original learning and interference learning. Recall included one cued recall trial without feedback for each set.

The second test session (Test session 2) started with recall of the original object-location sets (Recall original). The recall procedure and order of sets was the same as during the first test session. After recall of the original sets, the interference sets were tested with the same procedures (Recall Interference). It should be noted that performance in the second test session might be influenced by testing during the first test session (e.g., Karpicke and Roediger, 2008).

Learning level was calculated as the percentage of correctly recalled locations (out of 15 positions) in the last trial of the immediate cued recall trials of the learning session. Retention rate at the first test session was measured as the percentage of correctly recalled positions with the learning level set to 100%. Likewise, retention rate at the second test session was measured as the percentage of correctly recalled positions with the learning level set to 100%. Interference learning was measured as the percentage of correctly recalled locations (out of 15 positions) immediately after interference learning. Likewise, interference recall at the second test session was measured as the percentage of correctly recalled locations (out of 15 positions). Overall error rates were calculated as the percentage of wrongly recalled positions (out of 15 positions). Additionally, error rates for the original sets were calculated separately for interference errors (wrong recall of interfering position of the respective set) and random errors (any other wrongly recalled position).

### 2.4. Control tasks

In order to control for general alertness, a 5-min vigilance task (Diekelmann et al., 2013a) measuring reaction time (in ms) and error rates (in %), and the Stanford Sleepiness Scale (SSS) (Hoddes et al., 1972) were applied. In Experiment 1, both measures were taken during the learning session (before the object-location task), in test session 1 (after the object-location task) and in test session 2 (before the object-location task). In Experiment 2, the SSS was applied once during the learning session (before the object-location task), twice in test session 1 (before interference learning and after the object-location test) and once in test session 2 (between the object-location task and interference test). Vigilance was measured during the learning session (before the object-location task), in test session 1 (between interference learning and after the object-location test) and in test session 2 (between the object-location task and interference test).

For sleep participants of both experiments, polysomnography included electroencephalography (EEG) at sites C3 and C4, electromyography (EMG, left and right musculus mentalis) and two channels of electrooculography (EOG). Visual offline sleep scoring was done by a trained scorer according to Rechtschaffen and Kales (1986) and was verified by a second experienced scorer. In Experiment 2, online visual sleep scoring was additionally applied to detect SWS for TMR application.

In both experiments, expectancy ratings were assessed in the final questionnaire to ensure that the relevance instruction affected both groups comparably. At the end of test session 1, participants had to indicate to which extent they expected that they would receive the same reward for both objet-location sets (1 “not at all” to 5 “very much”). At the end of test session 2, they were asked to which extent they expected that both object-location sets would be tested again (1 “not at all” to 5 “very much”).

In Experiment 2, an odor detection and subjective evaluation task was performed before and after the learning session. This task contained 20 trials (50% odor), in which participants were asked to indicate whether or not the odor was present. Subsequently, they provided ratings for valence, familiarity, excitement, intensity and plunge on a scale from 0 (not at all) to 9 (very much). In the morning after the experimental night, participants were asked whether they believed that they had received the odor, placebo or whether they did not know. Certainty about their belief was assessed on a scale from 0 (not at all certain) to 4 (very certain). Nocturnal odor and placebo stimulations were counted and analyzed using FieldTrip toolbox for MATLAB.

### 2.5. Data analysis

Data analysis was realized with the software IBM SPSS Statistics 26. Retention rates, error rates, interference errors and random errors were analyzed using mixed ANOVAs with Group as between-subject factor (Experiment 1: sleep/wake, Experiment 2: cueing/placebo) and the within-subject factors Relevance (relevant/irrelevant) and Session (Experiment 1: after ~12 h/after 1 week, Experiment 2: after first SWS period/after ~1 week). These overall ANOVAs were followed up by two separate ANOVAs for the first test session (Experiment 1: after ~12 h, Experiment 2: after the first SWS period) and for the second test session (for both experiments after ~1 week), with Group as between-subject factor (Experiment 1: sleep/wake, Experiment 2: cueing/placebo) and the within-subject factor Relevance (relevant/irrelevant). Learning level, number of trials to achieve the learning level of 60%, interference learning (during first test session) and interference recall (during second test session) and error rates for different error types were analyzed using mixed ANOVAs with the same factors (between-subject factor: Group, within-subject factor: Relevance). Note that for interference recall, one data set was missing (Experiment 2, placebo group).

Subjective sleepiness of the SSS as well as reaction times and error rates of the vigilance task were analyzed using mixed ANOVAs with the factor Group as between-subject factor and the within-subject factor Session (learning session, test session 1 (in Experiment 2, SSS was assessed twice) and test session 2). Data from the odor detection task (correct detection and subjective evaluation of the odor) of Experiment 2 was analyzed by mixed ANOVAs with Group as between-subject factor (cueing/placebo) and Change as within-subject factor (before/after learning session). Note that one data set was missing for the odor detection task. The number of participants in Experiment 2 guessing whether they received odor or placebo or whether they did not know, was compared by means of Chi^2^ test. Certainty in the cueing and placebo group was compared by means of Mann-Whitney U test. The number of participants in the cueing and placebo group correctly guessing that they received the odor, was compared by means of Chi^2^ test.

As *post-hoc* tests for all ANOVAs of both experiments paired/unpaired *t*-tests and Mann-Whitney U tests/Wilkoxon signed rank tests were applied depending on whether normal distribution was given or not and on whether samples were dependent or independent. The same applies for group comparisons (sleep/wake and cueing/placebo) for the analysis of sleep data, number of stimulations during different sleep stages (Experiment 2, Bonferroni-corrected for multiple tests as indicated), certainty ratings about whether odor or placebo was applied (Experiment 2) and for expectancy ratings concerning the relevance instruction after test sessions 1 and 2. Correlations were calculated as Pearson's product moment correlation (r) or Kendal Tau (τ) depending on whether normal distribution was given.

For Experiment 1, formal sleep scoring was based on *n* = 26, since two data sets could not be scored according to formal criteria due to artifacts in EEG, EOG and/or EMG for one subject and due to artifacts in EOG and EMG in another subject. However, visual inspection of sleep quality and quantity was possible, which is why these data were included in behavioral analysis. Correlation with non-REM sleep stages were based on *n* = 27, since in one of the data sets with artifacts, EEG was intact which allowed staging of non-REM stages.

When appropriate, we tested (the lack of) differences between the groups by calculating Bayes factors (BF) using a Bayesian repeated measures ANOVA and Bayesian *post-hoc* tests (t-test and Mann-Whitney U test). For this analysis, we used the software JASP, Version 0.16.3.0 with default priors and selected a null model for comparison. All comparisons were two-sided and the level of significance was set to 0.05, the level of a statistical trend toward significance was set to 0.10. With regard to our a priori hypothesized effects, we explored statistical trends and ran planned *post-hoc* analyses in the case of non-significant overall interaction effects as detailed at the respective places.

## 3. Results

### 3.1. Experiment 1

#### 3.1.1. Object-location task

Table 1 shows the means and standard errors of the means (M ± SEM) of performance measures in the object-location task including the learning level, number of learning trials, retention rates, interference learning, interference recall and error rates after ~12 h (first test session) and after ~1 week (second test session) for the sleep and the wake group.

**Table 1:** Performance in the object-location task in Experiment 1.

	**Sleep**		**Wake**	
	**Relevant**	**Irrelevant**	**Relevant**	**Irrelevant**
**Learning**			
Correct %	67.64 ± 1.50	69.75 ± 1.65	69.29 ± 1.46	70.71 ± 1.56
Learning trials	2.50 ± 0.27	2.04 ± 0.22	2.46 ± 0.30	1.89 ± 0.23
**Recall after** ~**12 h**				
Retention rate	58.70 ± 5.23	44.50 ± 4.70	41.07 ± 3.64	34.49 ± 4.66
Error rate	60.95 ± 3.37	68.57 ± 3.79	71.67 ± 2.71	75.48 ± 3.40
Interference errors	7.86 ± 1.46	10.24 ± 2.01	10.00 ± 1.70	9.76 ± 2.48
Random errors	53.10 ± 3.15	58.33 ± 3.41	61.67 ± 2.29	65.71 ± 3.44
**Recall after** ~**1 week**				
Retention rate	53.42 ± 4.68	38.35 ± 3.98	37.30 ± 3.58	34.90 ± 4.02
Error rate	64.05 ± 3.19	72.86 ± 3.12	73.81 ± 2.74	75.24 ± 2.99
Interference errors	9.52 ± 1.76	9.76 ± 1.66	13.10 ± 2.73	14.29 ± 2.24
Random errors	54.52 ± 3.38	63.10 ± 3.15	60.71 ± 2.60	60.95 ± 2.72
**Interference learning after** ~**12 h**				
Correct %	42.18 ± 5.03	51.43 ± 4.55	59.46 ± 4.26	63.57 ± 4.52
Error rate	57.86 ± 5.03	48.57 ± 4.55	40.48 ± 4.27	36.43 ± 4.53
Interference errors	5.71 ± 1.27	4.29 ± 1.10	2.62 ± 0.79	2.14 ± 0.84
Random errors	52.15 ± 4.72	44.29 ± 4.31	37.86 ± 3.91	34.29 ± 4.31
**Interference recall after** ~**1 week**				
Correct %	17.00 ± 2.66	15.61 ± 2.64	18.00 ± 2.74	21.00 ± 2.96
Error rate	83.10 ± 2.67	84.05 ± 2.66	81.90 ± 2.74	79.05 ± 2.96
Interference errors	13.33 ± 1.94	13.81 ± 2.30	10.95 ± 1.85	8.57 ± 1.45
Random errors	69.76 ± 2.96	70.24 ± 2.80	70.95 ± 2.91	70.48 ± 3.04

Correct %: correctly recalled card locations (percentage out of 15) in the criterion trial of the learning session, interference learning and interference recall. Learning trials: number of repetitions needed to reach the learning criterion of 60%. Retention rate: percentage of correctly recalled card positions relative to the learning performance. Error rate: wrongly recalled card positions (percentage out of 15). Interference errors: percentage of errors related to the other version (i.e., interference task and original task, respectively). Random errors: percentage of errors not related to the other version. Means ± SEM are shown.

In the learning session, sleep and wake participants achieved comparable learning levels for the relevant and irrelevant sets, respectively, with 67.64 ± 1.50% and 69.75 ± 1.65% in the sleep group and 69.29 ± 1.46% and 70.71 ± 1.56% in the wake group (all *p* > 0.18, BF_01_ > 2.11). Participants needed on average a comparable number of trials to achieve the learning criterion of 60% for the relevant and irrelevant set, with 2.50 ± 0.27 and 2.04 ± 0.21 trials in the sleep group and 2.46 ± 0.30 and 1.89 ± 0.23 trials in the wake group, respectively (Group and interaction Group x Relevance: *p* > 0.74, BF_01_ > 4.04). Although the relevance instruction was only introduced after learning, participants required significantly more trials for learning of the relevant set than for the irrelevant set (Relevance: *p* = 0.039, η_p_^2^ = 0.076, BF_10_ = 1.95). However, the number of learning trials was not associated with retention rate (all r < 0.25, all *p* > 0.05).

**Figure 2:**
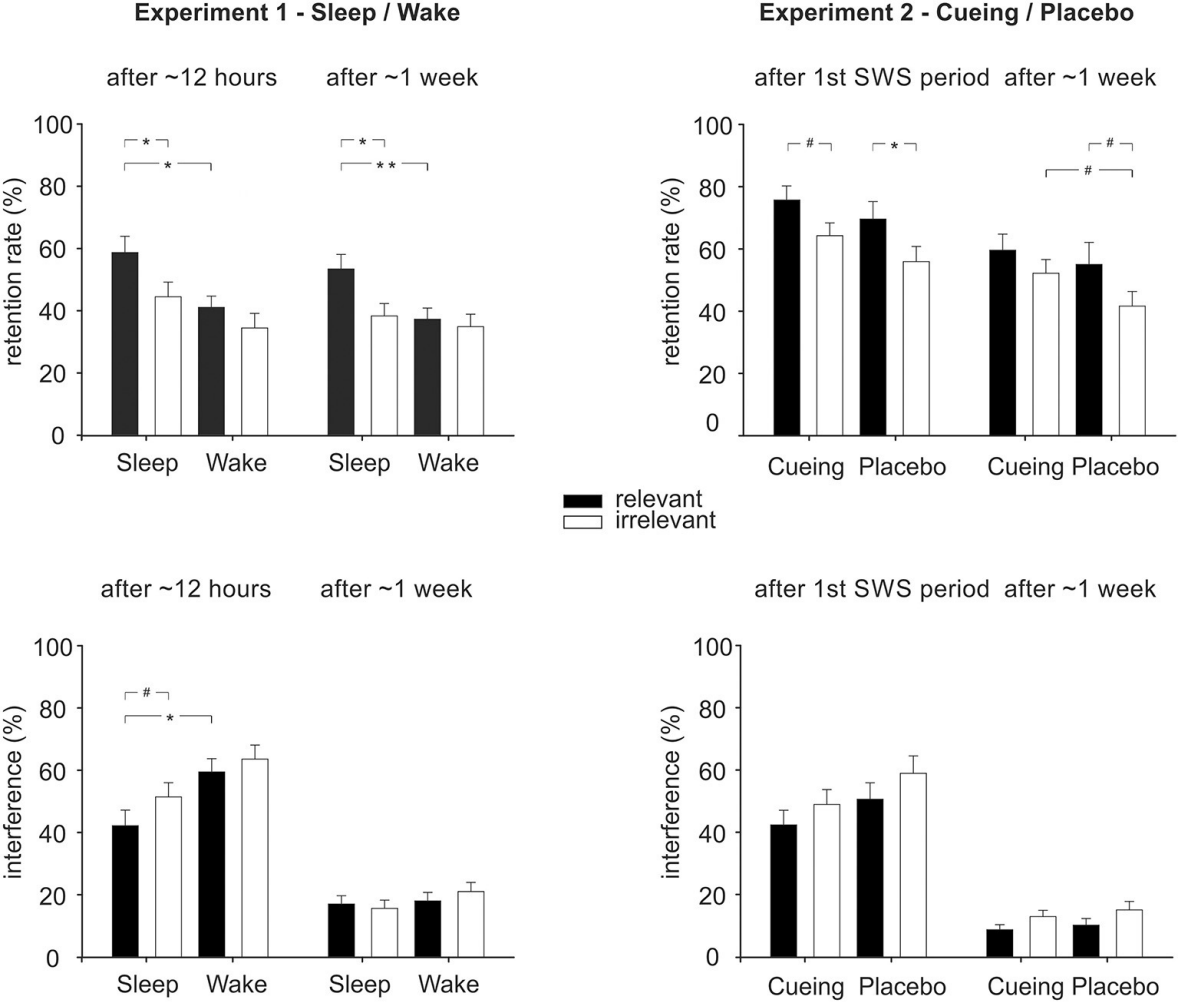
Memory performance in Experiment 1 (sleep/wake, left column) and Experiment 2 (cueing/placebo, right column) for the relevant and irrelevant sets of the original task (top row) and the interference task (bottom row). Performance is indicated as the retention rate, i.e., correctly recalled locations after the respective retention interval relative to the learning level (in %), as well as recall of interference locations (in % out of a total of 15). Performance is shown for the first test session (~12 h in Experiment 1, after the first SWS period in Experiment 2) as well as for the second test session (after ~one week in both experiments). *p < 0.05, **p < 0.01, ^#^*p* < 0.10.

Overall, retention rate was better when participants were allowed to sleep during the night after learning compared to participants who stayed awake [Figure 2, overall ANOVA Group: F_(1, 54)_ = 6.71, *p* = 0.012, η_p_^2^ = 0.11, BF_10_ = 4.16]. The sleep effect was evident both after ~12h [F_(1, 54)_ = 7.30, *p* = 0.009, η_p_^2^ = 0.12, BF_10_ = 4.13] and after ~one week [F_(1.54)_ = 4.95, *p* = 0.030, η_p_^2^ = 0.08, BF_10_ = 1.57], separately. Additionally, the relevant set was retained better than the irrelevant set [overall ANOVA: Relevance: F_(1, 54)_ = 8.78, *p* = 0.005, η_p_^2^ = 0.14, BF_10_ = 8.69]. This relevance effect was evident both after ~12h [F_(1, 54)_ = 6.76, *p* = 0.012, η_p_^2^ = 0.11, BF_10_ = 4.16] and after ~one week [F_(1, 54)_ = 5.47, *p* = 0.023, η_p_^2^ = 0.09, BF_10_ = 2.54]. Moreover, there was some evidence for a stronger effect of sleep on the consolidation of relevant memory. Although the interaction of Group x Relevance was not significant in the overall ANOVA (*p* = 0.12, BF_01_ = 0.03) as well as in the separate ANOVA after ~12 h (*p* = 0.35, BF_01_ = 0.14), there was a trend toward an interaction after ~1 week [Group x Relevance: F_(1, 54)_ = 2.87, *p* = 0.096, η_p_^2^ = 0.05, BF_01_ = 0.27]. *Post-hoc* analyses showed that after ~1 week, sleep participants retained the relevant set better than the irrelevant set [Figure 2, top left; *t*_(27)_ = 2.75, *p* = 0.01, BF_10_ =4.45], which was not the case for the wake participants [*t*_(27)_ = 0.47, *p* = 0.64, BF_01_ = 4.50]. Also, sleep participants retained the relevant set better than the wake participants [*t*_(54)_ = 2.74, *p* = 0.008, BF10 = 5.47], which was not the case for the irrelevant set [*t*_(54)_ = 0.61, *p* = 0.54, BF01 = 3.17]. This pattern was also already present after ~12h [Sleep: relevant > irrelevant, Z = −2.52, *p* = 0.012, BF_10_ = 4.966; Wake: *t*_(27)_ = 1.22, *p* =0.23, BF_01_ = 2.55; Relevant: sleep > wake, U = 251.00, Z = −2.31, *p* = 0.021, BF10 = 3.70; Irrelevant: *t*_(54)_ = 1.51, *p* = 0.14, BF_01_ = 1.44] and was further confirmed by a significant main effect of Relevance in the sleep group, with overall better retention for the relevant set after sleep [F_(1, 27)_ = 7.25, *p* = 0.01, BF_10Relevance_ = 3.98], while there was no such relevance effect in the wake group [F_(1, 27)_ = 1.66, *p* = 0.21, BF_01Relevance_ = 2.14] across both test sessions [Sleep: Relevance x Time: F_(1, 27)_ = 0.07, *p* = 0.80; Wake: Relevance x Time, F_(1, 27)_ = 0.28, *p* = 0.60]. Overall, the retention rate declined from the first to the second test session [Time: F_(1, 54)_ = 6.83, *p* = 0.012, η_p_^2^ =0.11, BF_10_ = 2.76; Time x Group: F_(1, 54)_ = 2.03, *p* = 0.16, Time x Group x Relevance: F_(1, 54)_ = 0.35, *p* = 0.70].

A similar pattern was obtained when analyzing error rates, but effects pointed in the inverse direction, i.e. less errors occurred after sleep than after wakefulness and less errors occurred in the relevant compared to the irrelevant set (see Table 1 for M ± SEM and Supplementary Table S1 for statistics). The reduction of error rates occurred to a similar degree in interference errors and random errors (Table 1, Supplementary Table S1).

Interference learning after ~12 h was impaired in participants who slept after encoding of the original memory when compared to participants who stayed awake [ANOVA after ~12 h, Group: F_(1, 54)_ = 8.42, *p* = 0.005, η_p_^2^ = 14, BF_10_ = 6.20]. Although the Group x Relevance interaction was not significant (*p* = 0.53, BF_01_ = 0.70), exploratory analyses pointed toward a stronger impairment in the sleep group compared to the wake group for interference learning of the relevant set (Figure 2, left bottom; relevant: *p* = 0.01, BF_10_ = 3.01; irrelevant: *p* = 0.10, BF_01_ = 1.10). There was also a trend toward a stronger impairment of interference learning for the relevant than for the irrelevant set in the sleep group (sleep: *p* = 0.094, BF_10_ = 0.97; wake: *p* = 0.632, BF_01_ = 3.78). Again, a similar pattern was obtained when analyzing error rates and when splitting error rates into interference errors and random errors (see Table 1 for M ± SEM and Supplementary Table S1 for statistics). Exploratory analyses of the association of learning and recall of the original task with learning and recall of the interference task revealed hints toward an inverse relationship at testing after ~12 h and after ~one week (see Supplementary Table S2).

#### 3.1.2. Sleep data and control tasks

**Figure 3:**
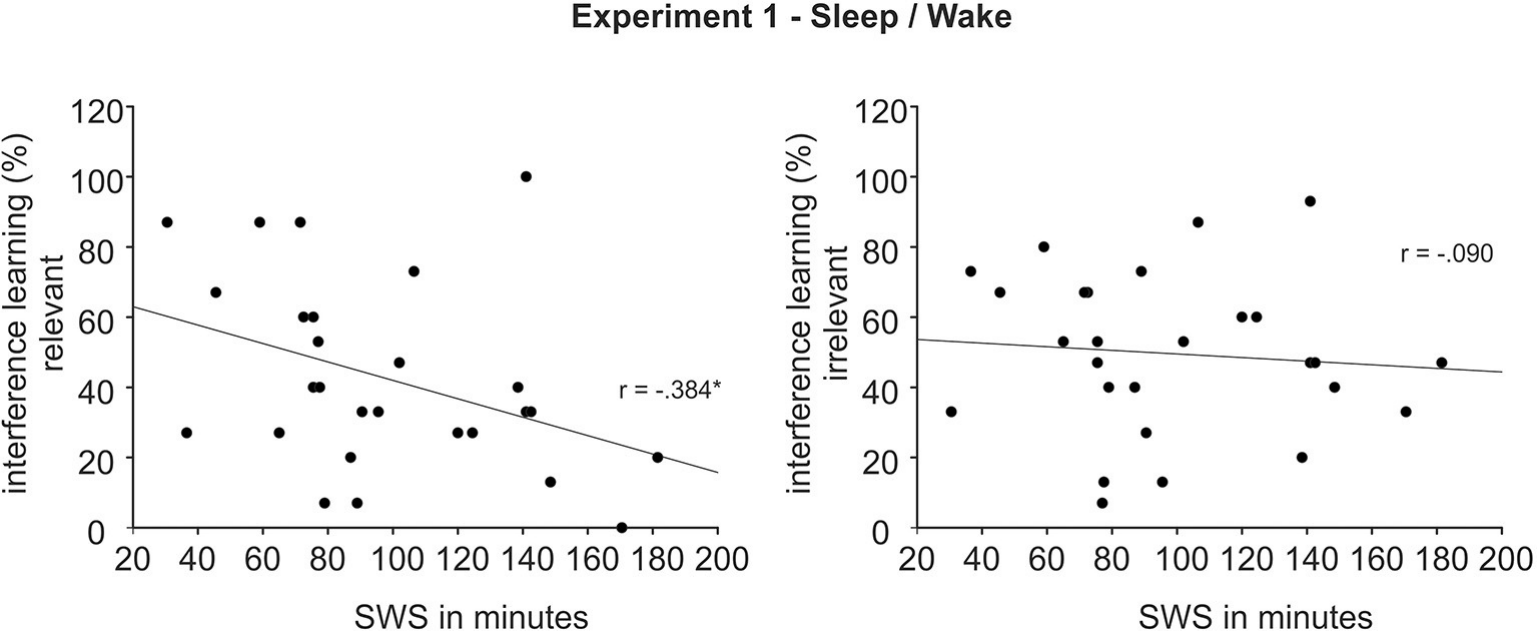
Association between time in Slow Wave Sleep (SWS) and interference learning after ~12 h in the sleep group of Experiment 1 for the relevant (**Left**) and irrelevant set (**Right**). *p < 0.05.

Sleep participants showed a normal sleep pattern. They slept on average 458.88 ± 34.37 min [total sleep time (TST); M ± SD], of which they spent 10.50 ± 7.80 min awake, 33.75 ± 19.76 min in S1, 213.56 ± 37.17 min in S2, 97.06 ± 40.21 min in SWS (S3: 51.17 ± 20.02, S4: 45.88 ± 28.38) and 99.29 ± 17.10 min in REM sleep. Participants fell asleep after on average 12.08 ± 10.37 min, the first epoch of SWS occurred on average after 16.08 ± 11.98 min and the first REM sleep epoch occurred on average after 85.58 ± 30.89 min. Interestingly, interference learning correlated with time spent in SWS for pictures of the relevant set. Longer SWS duration was associated with less successful interference learning for the relevant set (r = −0.38, *p* = 0.048, see Figure 3, **Left**), with no such correlation for the irrelevant set (r = −0.09, *p* = 0.65, see Figure 3, **Right**). The difference between these correlations showed a trend toward significance (z = −1.37, *p* = 0.086). There were no other correlations for TST, S2, SWS and REM sleep with learning of the original task and the interference task as well as with any of the retention rate measures, except for an association between S2 and interference recall of the relevant set after 1 week (r =0.29, *p* = 0.04; all other *p* > 0.05). Actigraphic recordings and daytime activity reports confirmed that none of the participants of the wake group slept during the day.

Sleep and wake participants were comparable in reaction times and error rates of the vigilance task (Group and Group x Time interaction: all *p* > 0.25, Table 2). However, sleep and wake participants differed in subjective sleepiness at the different time points (Group x Time interaction: *p* < 0.001, Table 2). During the learning session, sleep participants (for whom learning took place in the evening) were sleepier than wake participants (with learning taking place in the morning; U = 186.00, Z = −3.60, *p* < 0.001). However, sleepiness during the learning session was not associated with the learning level and the number of learning trials, neither with the retention rate after ~12 h and ~1 week (all *p* > 0.07). Sleep and wake groups did not differ in subjective sleepiness at the first test session after ~12 h as well as at the second test session after ~one week (all *p* > 0.30).

**Table 2:** Vigilance and subjective sleepiness in Experiment 1 and Experiment 2.

	**Experiment 1**		**Experiment 2**	
	**Sleep**	**Wake**	**Cueing**	**Placebo**
**Vigilance performance**				
***Reaction time (ms)***				
Learning session	369.67 ± 4.99	361.72 ± 6.91	434.37 ± 5.37	442.40 ± 12.33
After ~12h/1^st^ SWS period	380.38 ± 7.40	375.94 ± 9.18	459.43 ± 7.44	472.93 ± 14.39
After ~1 week	373.34 ± 5.54	376.96 ± 10.77	432.83 ± 6.89	456.40 ± 14.17
***Error rate ( %)***				
Learning session	3.04 ± 0.63	2.50 ± 0.41	3.15 ± 0.96	1.75 ± 0.48
After ~12h/1^st^ SWS period	3.89 ± 0.59	2.87 ± 0.44	1.63 ± 0.51	1.50 ± 0.58
After ~1 week	3.66 ± 0.41	3.39 ± 0.68	2.50 ± 0.47	3.13 ± 0.57
**Subjective sleepiness**				
Learning session	3.07 ± 0.19	2.18 ± 0.12	3.00 ± 0.19	2.90 ± 0.19
Before interference learning	-	-	3.70 ± 0.28	3.90 ± 0.22
After ~12h/1^st^ SWS period	2.21 ± 0.15	2.54 ± 0.22	3.61 ± 0.30	2.55 ± 0.27
After ~1 week	2.64 ± 0.19	2.82 ± 0.20	2.43 ± 0.15	2.70 ± 0.24

Means ± SEM are shown.

Participants' expectation that both card-pair sets would be tested and rewarded after ~12h was relatively low (sleep: 2.75 ± 0.28, wake: 2.11 ± 0.26), whereas after ~one week, expectation that both card-pair sets would be tested again was rather high (sleep: 4.00 ± 0.22, wake: 4.11 ± 0.22). Expectancy ratings did not differ between groups (p >0.10 for both time points).

### 3.2. Experiment 2

#### 3.2.1. Object-location task

Table 3 shows means and standard errors of the means (M ± SEM) of performance measures in the object-location task including the learning level, number of learning trials, interference learning, recall, interference recall and error rates after the first SWS period (first test session) and after ~1 week (second test session) for the cueing and the placebo group, respectively.

**Table 3:** Performance in the object-location task in Experiment 2.

	**Cueing**		**Placebo**	
	**Relevant**	**Irrelevant**	**Relevant**	**Irrelevant**
**Learning**				
Correct %	72.78 ± 2.09	71.39 ± 2.45	72.95 ± 2.19	72.25 ± 2.82
Learning trials	2.13 ± 0.33	2.39 ± 0.23	1.85 ± 0.20	1.85 ± 0.20
**Recall after 1st SWS period**				
Retention rate	75.71 ± 4.51	64.25 ± 4.11	69.58 ± 5.62	55.93 ± 4.88
Error rate	44.35 ± 3.74	53.62 ± 3.47	48.67 ± 4.77	59.00 ±4.33
Interference errors	6.67 ± 2.05	8.41 ± 1.58	8.00 ± 1.97	12.33 ± 2.99
Random errors	37.68 ±3.47	45.22 ± 3.38	40.66 ± 4.10	46.67 ± 3.93
**Recall after** ~**1 week**				
Retention rate	59.59 ± 5.18	52.22 ± 4.39	55.02 ± 7.10	41.65 ± 4.69
Error rate	56.81 ± 3.60	62.90 ± 3.27	59.67 ± 5.35	70.00 ± 3.44
Interference errors	6.96 ± 1.42	5.80 ± 1.21	7.67 ± 1.47	6.00 ± 1.17
Random errors	51.01 ± 3.04	55.94 ± 3.04	53.67 ± 5.26	62.33 ± 3.75
**Interference learning after** ~**12 h**				
Correct %	41.43 ± 4.72	49.00 ± 4.79	50.70 ± 5.25	59.00 ± 5.60
Error rate	58.55 ± 4.72	51.01 ± 4.79	49.33 ± 5.24	41.00 ± 5.60
Interference errors	5.80 ± 2.15	5.80 ± 1.28	3.33 ± 1.41	3.67 ± 1.23
Random errors	52.76 ± 3.84	45.22 ± 4.23	46.00 ± 4.61	37.33 ± 5.10
**Interference recall after** ~**1 week**				
Correct %	8.77 ± 1.59	12.97 ± 1.99	10.23 ± 2.11	15.11 ± 2.72
Error rate	91.30 ± 1.59	86.96 ± 1.99	89.82 ± 2.12	84.91 ± 2.74
Interference errors	11.30 ± 3.12	10.72 ± 2.61	10.88 ± 2.51	10.18 ± 1.86
Random errors	80.00 ± 2.78	76.23 ± 2.54	78.95 ± 3.43	74.74 ± 3.63

Correct %: correctly recalled card locations (percentage out of 15) in the criterion trial of the learning session, interference learning and interference recall. Learning trials: number of repetitions needed to reach the learning criterion of 60%. Retention rate: percentage of correctly recalled card positions relative to the learning performance. Error rate: wrongly recalled card positions (percentage out of 15). Interference errors: percentage of errors related to the other version (i.e. interference task and original task, respectively). Random errors: percentage of errors not related to the other version. Means ± SEM are shown.

In the learning session, participants of the cueing and the placebo group achieved comparable learning levels, with 72.78 ± 2.09% and 71.39 ± 2.45% for the relevant and irrelevant set in the cueing group and 72.95 ± 2.19% and 72.25 ± 2.28% in the placebo group (all *p* > 0.59, BF_01_ > 3.30). Participants of the cueing and placebo group also needed a comparable number of trials to achieve the learning criterion of 60% with 2.13 ± 0.33 and 2.39 ± 0.23 trials for the relevant and irrelevant set in the cueing group and 1.85 ± 0.20 and 1.85 ± 0.20 in the placebo group (all *p* > 0.16, BF_01_ > 1.50).

Overall, the retention rate after odor cueing was not significantly different from placebo, neither when testing took place after the first SWS period nor after ~1 week [Group: F_(1, 41)_ = 2.10, *p* = 0.16, BF_01_ = 1.22, for overall ANOVA; F(1,41) = 2.06*, p* = 0.16, BF_01_ = 1.84, for ANOVA after the first SWS period; F_(1, 41)_ = 1.43, *p* = 0.24, BF_01_ = 1.87, for ANOVA after ~1 week; see Table 3, Figure 2, top right]. As in Experiment 1, the relevant set was overall retained better than the irrelevant set [Relevance: F_(1, 41)_ = 9.04, *p* = 0.005, η_p_^2^ = 0.18, BF_10_ = 10.06] both after the first SWS period [F_(1, 41)_ = 7,85, *p* = 0.008, η_p_^2^ = 0.16, BF_10_ = 9.54] and after ~1 week [F_(1, 41)_ = 5.98, *p* = 0.019, η_p_^2^ = 0.13, BF_10_ =2.64].

Although there was no evidence for an interaction between Group and Relevance (all interactions including Group and Relevance: *p* > 0.48, BF_01_ > 0.001, all other interactions: *p* > 0.60, BF_01_ ≈ 0), we ran planned *post-hoc* pairwise comparisons that revealed a marginally stronger increase in recall of the irrelevant set in the cueing group after ~one week (*p* = 0.073, BF_10_ = 1.47), which was not evident for the relevant set (*p* = 0.33, BF_01_ = 2.89). Additionally, at the ~one week recall, memory for the relevant set was retained marginally better than for the irrelevant set in the placebo group (z = −1.72, *p* = 0.086, BF_01_ = 0.62) but not in the cueing group (z = −1.16, *p* = 0.24, BF_01_ = 2.13). At the first test session after the first SWS period, there was no trend toward a differential cueing effect for the relevant and irrelevant sets (all *p* > 0.19, BF_01_ > 1.67). Participants showed significantly better retention of the relevant set compared to the irrelevant set in the placebo group [*t*(19) = 2.12, *p* = 0.048, BF_10_ = 1.44] and a trend in the same direction for the cueing group [*t*_(22)_ = 1.85, *p* = 0.078, BF_01_ = 1.07]. Overall, retention declined from the first test session to after ~1 week [Time: F_(1, 41)_ = 30.73, *p* < 0.001, η_p_^2^ = 0.50, BF_10_ = 7,219.61], which was evident in all *post-hoc* pairwise comparisons (all *p* < 0.045).

Similar patterns of results were obtained when analyzing error rates including interference errors and random error types (see Table 3 for M ± SEM and Supplementary Table S3 for statistics).

Odor cueing did not affect interference learning during the first test session [Group: *p* = 0.138, BF_01_ = 1.16] nor interference recall after ~1 week (Group: *p* = 0.41, BF_01_ = 3.00). Overall, interference learning for the relevant set was less successful than for the irrelevant set, both after the first period of SWS [Relevance: F_(1, 41)_ = 5.75, *p* = 0.021, η_p_^2^ = 0.123, BF_10_ = 2.56] and after ~1 week [Relevance: F_(1, 41)_ = 4.90, *p* = 0.033, η_p_^2^ = 0.109, BF_10_ = 2.82]. This effect was independent of odor cueing (interactions Group x Relevance: all *p* > 0.86, see Figure 2, bottom right). Similar patterns of results were obtained when analyzing error rates including interference errors and random error types (Table 3, Supplementary Table S3). Exploratory analyses of the association of learning and recall of the original task with learning and recall of the interference task revealed no consistent results (see Supplementary Table S2).

#### 3.2.2. Sleep data and control tasks

Participants in the cueing and the placebo group showed comparable sleep patterns during the first sleep period as well as during the rest of the night (Table 4). The number of cueing events in different sleep stages was also comparable between the cueing and placebo groups (all *p* > 0.05, Bonferroni-corrected for 6 tests). Overall, participants of the cueing group received 31.70 ± 12.50 cueing events and participants of the placebo group received 39.20 ± 13.37 cueing events [*t*_(41)_ = 1.90, p_6_ = 0.38], of which the majority was applied during SWS (Cueing: 28.43 ± 12.95, Placebo: 34.55 ± 13.42, U = 161.00, Z = −1.68, p_6_ = 0.56). There were no correlations of sleep data during the first SWS period (i.e., TST, S2, SWS; there was no REM sleep) with learning of the original task and the interference task as well as with any of the retention rate measures, except for a negative association between S2 and the retention rate for the relevant set after 1 week in the placebo group (r = −0.47, *p* = 0.04; all other *p* > 0.05).

**Table 4:** Sleep parameters in Experiment 2.

	**1st SWS period**		**Rest of the night**	
	**Cueing**	**Placebo**	**Cueing**	**Placebo**
TST	55.28 ± 19.76	56.03 ± 15.17	378.11 ± 30.07	371.18 ± 27.74
W	2.61 ± 5.47	0.83 ± 2.38	4.15 ± 4.49	7.90 ± 8.27
S1	5.89 ± 4.03	4.90 ± 3.61	22.78 ± 8.67	24.38 ± 10.21
S2	17.98 ± 7.31	15.83 ± 5.01	190.93 ± 28.23	195.60 ± 23.96
S3	13.13 ± 9.80	21.63 ± 11.82	41.83 ± 15.12	39.63 ± 11.39
S4	15.57 ± 10.97	12.78 ± 12.54	19.09 ± 15.19	8.88 ± 11.05
SWS	28.70 ± 12.73	34.40 ± 13.10	60.91 ± 22.05	48.50 ± 18.96
REM	-	-	97.17 ± 15.70	92.78 ± 14.69
Sleep latency	14.36 ± 9.05	12.85 ± 9.75	16.57 ± 9.52	17.27 ± 11.55
SWS latency	22.02 ± 11.99	17.33 ± 6.60	33.57 ± 19.65	24.40 ± 10.69
REM latency	-	-	55.89 ± 19.80	60.28 ± 14.94

TST, Total Sleep Time; W, time awake after sleep onset; S1–S4, sleep stages 1–4; SWS, slow wave sleep; REM, rapid eye movement sleep; Sleep latency, time to the first S1 epoch followed by S2; SWS latency, time to first SWS epoch; REM latency, time to first REM epoch. Means ± SD are shown in minutes.

Cueing and placebo participants did not differ in reaction times and error rates of the vigilance task (all *p* > 0.15), as well as in subjective sleepiness (all *p* > 0.70, Table 2). All participants reliably distinguished the odor substance from placebo with 91.91 ± 1.09% correct responses in the odor detection task averaged over the test immediately before and after the learning session. Correct detections were comparable between cueing and placebo participants and before and after the learning session (all *p* > 0.24). The odor was evaluated as neither positive nor negative (M = 5.14 ± 0.25), slightly unfamiliar (M = 4.60 ± 0.35), rather unexciting (3.52 ± 0.43), slightly intense (5.75 ± 0.24), and rather not pungent (3.71 ± 0.32). These evaluations were comparable between cueing and placebo participants (all *p* >0.08). When asked about which substance they had received during the experimental night, 41.9% of the participants selected the option “I don't know,” 34.9% selected “placebo” and 23.2% selected “odor” (Chi^2^ = 0.26, *p* = 0.88, for group comparison). Cueing and placebo groups did not differ in the number of correct guesses (Chi^2^(1) = 1.07, *p* = 0.43) nor in their certainty (U = 132.00, z = −1.13, *p* = 0.26).

Participants' expectation that both card-pair sets would be tested and rewarded after the first SWS period was rather low (cueing: 1.89 ± 0.23, placebo: 1.89 ± 0.24). After ~11 week, expectation that both card-pair sets would be tested again was higher (cueing: 3.7 ± 0.30, placebo: 3.00 ± 0.03). At both time points, cueing and placebo participants did not differ in their expectation (*p* > 0.10).

## 4. Discussion

We examined in two experiments the effects of sleep, relevance, targeted memory reactivation (TMR) and delay on memory consolidation. Experiment 1 confirmed the expected better memory retention after sleep compared to wakefulness (main effect sleep). Relevant information was also overall better retained than irrelevant information (main effect relevance). Although overall, memory declined over the course of 1 week (main effect time), the beneficial main effects of sleep and relevance persisted after 1 week. Moreover, there was tentative evidence that relevant information is preferentially consolidated during sleep when compared to irrelevant information, with this effect becoming more prominent after 1 week (although the interaction did not reach significance). Experiment 2 replicated the preferential consolidation of relevant over irrelevant memories during sleep (main effect relevance) as well as the general memory decline over the course of 1 week (main effect time), with the advantage for relevant memories persisting after 1 week. Contrary to our hypothesis, TMR did not preferentially facilitate the consolidation of relevant memories, neither after the first SWS period nor after 1 week.

The beneficial effect of sleep for memory observed in Experiment 1 confirmed and replicated previous evidence of a general improvement of the consolidation of declarative memories after sleep compared to wakefulness (Walker and Stickgold, 2006; Rasch and Born, 2013; Tononi and Cirelli, 2014). Following a night of sleep, participants retained more of the encoded information, committed overall fewer errors, and showed impairments in interference learning after sleep, suggesting that sleep-dependent consolidation stabilized the newly encoded memories. Additionally, relevant memories were overall better retained than irrelevant memories, which is in line with previous findings (Zeigarnik, 1927; Mäntyl and Sgaramella, 1997; Walter and Meier, 2014, 2017).

Experiment 1 further provided tentative evidence for our hypothesis of a preferential consolidation of relevant over irrelevant information during sleep. Although the overall interaction effect was not significant, planned *post-hoc* comparisons revealed that better retention of relevant over irrelevant memories was stronger after sleep compared to wakefulness. This effect was already evident after 12 h but became even more pronounced after 1 week. The finding that sleep preferentially consolidates relevant memories has previously been observed after a nap (Bennion et al., 2016) and after one night of sleep (Fischer and Born, 2009; Wilhelm et al., 2011; Van Dongen et al., 2012b) but the persistence of this effect over longer time periods has not been shown before. We suggest that the effect observed in the present study relies on a process of stabilization and active system consolidation mainly during the first night of sleep after encoding, preferentially for the relevant material. This interpretation is supported by the finding that sleep participants were impaired in interference learning after the first night especially for information which interfered with the relevant materials. Such an impairment of interference learning as a measure of memory stabilization has previously been reported already after 40 min of sleep, rich in SWS (Diekelmann et al., 2012), which highlights the potential role of SWS for the stabilization of memories. Interestingly, in the present study, longer duration of SWS was associated with stronger impairments in interference learning specifically for relevant items but not for irrelevant ones, indicating that SWS-related processes may be particularly implicated in the preferential consolidation of relevant over irrelevant materials. It could be speculated that relevant memories become tagged during encoding (Ballarini et al., 2009; Dunsmoor et al., 2015; Moncada et al., 2015), with this tagging allowing for a preferential access of these memories to consolidation processes during SWS.

The finding that the advantage of relevant over irrelevant memories after sleep may become even stronger 1 week later is particularly interesting. Only few studies have investigated longer retention intervals for sleep-dependent memory consolidation. Some studies observed benefits of sleep in the declarative domain after 1 week (Cousins et al., 2019), and even after 1 year for the abstraction of gist memory (Lutz et al., 2017), while others found no persistent sleep benefits after 1 week (Abel et al., 2019). The present study suggests that the benefits of sleep for memory consolidation may only persist for information that is relevant for the individual but not for irrelevant information memories (Wagner et al., 2006; Bolinger et al., 2019). Indeed, the data of the present study indicate that after sleep, irrelevant memories were subject to forgetting to a stronger degree than relevant memories. The first night of sleep after encoding may play a particular role in this long-term effect. Considering that the instruction for retrieval specifically for the relevant memories was revoked before the first test session and not renewed thereafter, and thus, the relevance instruction was only active during the first night of sleep, the persisting and even increasing advantage of relevant over irrelevant memories after 1 week suggests that this was mainly a result of preferential stabilization and system consolidation of relevant materials during the first night of sleep after encoding. Alternatively, the stronger relevance effect for sleep-dependent consolidation after 1 week could at least partly be explained by a testing effect from the first test session. It is well-known that testing strengthens memories through retrieval practice (Karpicke and Roediger, 2008; Rowland, 2014; Adesope et al., 2017). Considering that relevant memories were recalled better than irrelevant memories during the first test session in the present study (even slightly more so in the sleep group), relevant memories (especially in the sleep group) might have benefitted more from the testing effect, resulting in a stronger difference between relevant and irrelevant memories in the sleep group 1 week later. However, recent evidence suggests that sleep may rather reduce the testing effect, leaving open this question for further investigation (Abel et al., 2019).

The findings of Experiment 2 confirm those of Experiment 1 in showing that relevant information is consolidated more successfully during sleep, such that even after a short period of 40 min of sleep, memories for relevant materials are retained better, are less prone to errors, and are less susceptible to interference learning than memories of irrelevant information. However, contrary to our hypothesis, TMR with odor cueing did not facilitate memory consolidation in general, neither specifically for the relevant material. We had originally hypothesized that the simultaneous cueing of relevant and irrelevant memories by a context cue like an odor would induce (or foster) the competition between relevant and irrelevant memories for potentially limited reactivation capacities (Bendor and Wilson, 2012; Feld et al., 2016; Antony et al., 2018). The present findings do not support this hypothesis. On the contrary, the data, if at all, even point to a slightly stronger benefit of cueing for the consolidation of irrelevant information, which would be in line with previous findings from Oudiette et al. (2013).

Several reasons might account for the lack of an overall TMR effect as well as for the lack of a selective relevance-specific TMR effect. First, the study was possibly underpowered to detect a potentially small interaction effect, due to a relatively small sample size. Furthermore, the performance level at the first test session after 40 min of sleep was relatively high compared to that after one night of sleep in Experiment 1, such that a ceiling effect might have limited the space for further improvements by TMR. Moreover, the type of relevance manipulation in the present study might have constituted a very strong top-down signal for the access to endogenous reactivation processes. In the present study, we combined the announcement of a retrieval test after sleep with the announcement of a monetary reward for good performance at retrieval for the induction of relevance. Both of these manipulations have been shown to induce a solid advantage of relevant over irrelevant memories for sleep-related consolidation when applied separately (Fischer and Born, 2009; Wilhelm et al., 2011; Van Dongen et al., 2012b) and even more so when applied in combination (see Bennion et al., 2016) as well as Experiment 1 of the present study). It has been suggested that different means of relevance induction vary in their effectiveness of signaling relevance for long-term consolidation. While task-inherent bottom-up features, like the salience or the emotional valence of an item, seem to be less effective, peri-encoding or post-encoding top-down relevance signals, like the announcement of a reward or a retrieval test, produce stronger advantages of relevant over irrelevant materials (Bennion et al., 2016). As a consequence, a relatively strong relevance instruction in the present study, by combining two top-down relevance signals, might have induced robust spontaneous reactivation during sleep, such that relevant memories might have already received the maximally possible reactivation capacities. Alternatively, or additionally, the bottom-up external cueing signal might have been too weak to compete with the stronger top-town relevance signals in the competition for access to reactivation resources. Thus, strong endogenous reactivation processes might trump the potentially weaker effect of external cued reactivation. Importantly, this explanation might still allow for a selective TMR benefit for irrelevant (or low-reward memories) as shown by Oudiette et al. (2013) as well as descriptively in the present study, considering that these memories would not benefit from the strong top-down relevance signals and might therefore be susceptible to a facilitation of reactivation by external cueing. Thus, potential effects of TMR might have been masked by ceiling effects and/or strong endogenous reactivation in the present study. This should be further explored in future studies.

With regard to the time course of memory consolidation, Experiment 2 confirmed our findings from Experiment 1, in showing that memory overall deteriorated over time but the advantage of relevant over irrelevant information persisted at the 1 week retrieval test. It could even be speculated that potential effects of TMR might become even stronger after 1 week. Although none of the effects reached significance, the unexpected descriptive improvement of irrelevant materials by TMR was stronger at the second test session after 1 week. Together with Experiment 1 showing that the advantage of relevant over irrelevant memories after sleep becomes slightly stronger after 11 week, these data may indicate a persistent and potentially even increasing long-term effect of sleep-dependent memory consolidation. Long-term effects should be more prominently investigated in future studies.

Several limitations should be considered with regard to the present findings. First, considering the experimental design of Experiment 1, with encoding and retrieval test sessions taking place at different times of the day for sleep and wake participants, differences due to circadian effects cannot be excluded. However, the encoding level, number of trials to reach the learning criterion, as well as objective vigilance measures did not differ between groups. Only subjective sleepiness was higher in the sleep group during encoding, but sleepiness ratings were not associated with any of the learning and retrieval measures, speaking against substantial circadian confounds. Second, the relevance instruction was given only after encoding, i.e., with a certain delay, in order to disentangle effects on encoding and consolidation, which is in line with most studies using top-down relevance instructions in sleep and memory consolidation studies (Fischer and Born, 2009; Wilhelm et al., 2011; Van Dongen et al., 2012b; Diekelmann et al., 2013b; see e.g. Cunningham et al., 2014; Wamsley et al., 2016; Reverberi et al., 2020). However, others have applied the relevance instruction during encoding on the basis of single items (e.g., Bennion et al., 2016), which might yield an overall stronger tagging effect for relevant items and could potentially induce a stronger selectivity of sleep-dependent consolidation for relevant over irrelevant information. Third, the second test session was influenced by recall of the material during the first test session. A possible testing effect might have changed the long-term effect of sleep for the relevant memories at the second test session after 1 week (e.g., Karpicke and Roediger, 2008; Abel et al., 2019). Thus, the findings after 1 week should therefore be interpreted with caution. Fourth, the sleep-specific relevance effect might be smaller than previous studies suggest (Cordi and Rasch, 2021). Although our study was powered to detect medium effect sizes, the power was not sufficient to detect small effect sizes. Considering that other studies failed to replicate the relevance-driven consolidation effect during sleep (Wamsley et al., 2016; Reverberi et al., 2020), this effect might indeed be smaller than the overall sleep effect for memory in general. In line with this hypothesis, the present findings showed a strong and consistent sleep effect for memory overall, but only a weak (and non-significant) interaction effect for the preferential consolidation of relevant information during sleep. It is important to note that several comparisons in the present study did not reach significance and, thus, the present findings should be interpreted with caution and await further confirmation. Fifth, a general problem of studies on preferential memory consolidation during sleep is that the experimental relevance manipulations may induce differences in memory strength already during the encoding phase before sleep. In the present study, we have tried to minimize such effects as best as possible by introducing the relevance instruction only after the actual encoding phase. Nevertheless, it might be possible that this instruction changes memory strength during early consolidation processes before sleep. Future studies should consider testing differences in memory strength between relevant and irrelevant memories before sleep. Finally, we did not observe any consistent effects of TMR on relevant and irrelevant memories, as discussed above, which should be subject to further investigation.

## Data availability statement

The raw data supporting the conclusions of this article will be made available by the authors, without undue reservation.

## Ethics statement

The studies involving human participants were reviewed and approved by Ethics Committee of the Medical Faculty of the University of Tübingen. The patients/participants provided their written informed consent to participate in this study.

## Author contributions

CB designed the research, supervised data collection and data organization, analyzed the data, and wrote the manuscript. A-SW and SS contributed to the design of the research, collected the data, organized the data, and analyzed polysomnographic recordings. JB acquired the fund for this study, designed the research, and edited the manuscript. SD acquired the fund for this study, designed the research, analyzed the data, and edited the manuscript. All authors approved the submitted version.
