# Supplementary material for: The effects of sleep and targeted memory reactivation on the consolidation of relevant and irrelevant information
Source: Front Sleep. 2023 May 25;2:1187170. doi: 10.3389/frsle.2023.1187170 (PMC12713986; doi:10.3389/frsle.2023.1187170)
Supplement: Supplementary file 1 [file Table_1.DOCX]

Supplementary Material

The effects of sleep and targeted memory reactivation on the 
consolidation of relevant and irrelevant information

Christine Barner, Ann-Sophie Werner, Sandra Schörk, Jan Born, Susanne Diekelmann*

*** Correspondence:** Susanne Diekelmann (susanne.diekelmann@uni-tuebingen.de)

**Supplementary Table S1**. Statistics for error rates of the object-location task in Experiment 1

|  | p | ƞ_p_² | BF | | p | ƞ_p_² | | BF | | p | ƞ_p_² | | BF |
| --- | --- | --- | --- | --- | --- | --- | --- | --- | --- | --- | --- | --- | --- |
|  | Total error | | | Interference error | | | | | | Random error | | | |
| Original task |  |  |  | |  |  | |  | |  |  | |  |
| Overall ANOVA |  |  |  | |  |  | |  | |  |  | |  |
| Group | **.032** | .08 | 2.05 | | .205 | .03 | | 2.12 | | .102 | .05 | | 1.08 |
| Relevance | **.027** | .09 | 2.20 | | .450 | .002 | | 4.70 | | *.055* | .07 | | 0.79 |
| Time | **.021** | .10 | 0.62 | | *.056* | .07 | | 1.72 | | .950 | <.001 | | 5.48 |
| Time x Relevance | .840 | .001 | 3.72 | | .905 | <.001 | | 44.25 | | .927 | <.001 | | 20.39 |
| Time x Group | .166 | .04 | 2.41 | | .160 | .04 | | 8.84 | | *.065* | .061 | | 5.70 |
| Relevance x Group | .245 | .03 | 0.37 | | .724 | .002 | | 43.11 | | .307 | .02 | | 1.67 |
| Group x Relevance x Time | .544 | .01 | 21.75 | | .551 | .007 | | 2621.75 | | .171 | .03 | | 76.11 |
| After ~12h |  |  |  | |  |  | |  | |  |  | |  |
| Group | **.023** | .09 | 2.22 | | .690 | .003 | | 3.84 | | **.021** | .10 | | 0.47 |
| Relevance | *.051* | .07 | 0.81 | | .567 | 01 | | .422 | | .109 | .05 | | 1.41 |
| Relevance x Group | .51 | .01 | 1.09 | | .484 | .01 | | 48.82 | | .835 | .001 | | 2.40 |
| After ~1 week |  |  |  | |  |  | |  | |  |  | |  |
| Group | *.070* | .06 | 1.10 | | *.089* | .05 | | 1.24 | | .559 | .01 | | 3.26 |
| Relevance | *.066* | .06 | 0.96 | | .713 | .003 | | 4.56 | | *.074* | .06 | | 1.15 |
| Relevance x Group | .181 | .03 | 1.71 | | .806 | .001 | | 20.93 | | *.091* | .05 | | 4.04 |
| Interference task |  | | | | | | | | | | | | |
| Interference learning  after ~12h | | | |  | | |  | |  |  |  |  | |
| Group | **.005** | .14 | 6.46 | | **.017** | .10 | | 2.16 | | **.015** | .11 | | 3.01 |
| Relevance | .108 | .05 | 1.44 | | .333 | .02 | | 3.10 | | .136 | .04 | | 1.77 |
| Relevance x Group | .523 | .01 | 0.65 | | .627 | .004 | | 4.74 | | .57 | .006 | | 1.66 |
| Interference recall  after ~1 week | | | |  | | |  | |  |  |  |  | |
| Group | .281 | .02 | 2.68 | | *.067* | .06 | | 1.08 | | .820 | .001 | | 3.94 |
| Relevance | .724 | .002 | 4.78 | | *.059* | .005 | | 4.46 | | >.999 | <.000 | | 5.00 |
| Relevance x Group | .480 | .01 | 38.26 | | .424 | .01 | | 13.87 | | .861 | .001 | | 73.54 |

BF: BF_10_ for p < .05, BF_01_ for p > .05; Factor “Group”: sleep/wake, Factor “Relevance”: relevant/irrelevant, Factor “Time”: after ~12h/after ~1 week. Grey column highlights p values, significances are indicated in bold, trends are indicated in italic.

**Supplementary Table S2.** Associations between original and interference memory

| **Experiment 1** | **Overall (n = 56)** | | **Sleep (n = 28)** | | **Wake (n = 28)** | |
| --- | --- | --- | --- | --- | --- | --- |
|  | relevant | irrelevant | relevant | irrelevant | relevant | irrelevant |
| Learning original x learning interference (12 hrs apart) | 0.169 | **0.335** | **0.401** | 0.164 | -0.172 | **0.508** |
| Recall 1 original x learning interference (both at 12 hrs) | **-0.305** | -0.107 | -0.075 | -0.217 | **-0.436** | 0.105 |
| Recall 2 original x recall interference (both at 1 week) | **-0.281** | -0.114 | -0.174 | -0.005 | **-0.411** | -0.194 |
| **Experiment 2** | **Overall (n = 43)** | | **Cueing (n = 23)** | | **Placebo (n = 20)** | |
|  | relevant | irrelevant | relevant | irrelevant | relevant | irrelevant |
| Learning original x learning interference (3.5 hrs apart) | -0.083 | **0.346** | -0.032 | **0.487** | -0.150 | 0.178 |
| Recall 1 original x learning interference (both at 3.5 hrs) | -0.120 | 0.108 | -0.032 | 0.274 | -0.166 | 0.023 |
| Recall 2 original x recall interference (both at 1 week) | 0.098 | 0.058 | -0.027 | 0.226 | 0.199 | -0.056 |
| Values represent Pearson’s correlation coefficients *r* for percentage correct responses at the different time points and for the different materials. Significant correlations with p < .05 (uncorrected) are indicated in bold. After Bonferroni correction for multiple comparisons, none of the correlations remain significant. | | | | | | |

**Supplementary Table S3**. Statistics for error rates of the object-location task in Experiment 2

|  | p | ƞ_p_² | BF | p | ƞ_p_² | BF | p | ƞ_p_² | BF |
| --- | --- | --- | --- | --- | --- | --- | --- | --- | --- |
|  | Total error | | | Interference error | | | Random error | | |
| Original task |  |  |  |  |  |  |  |  |  |
| Overall ANOVA |  |  |  |  |  |  |  |  |  |
| Group | .225 | .04 | 1.53 | .331 | .02 | 2.79 | .340 | .02 | 3.67 |
| Relevance | **.004** | .18 | 10.14 | .482 | .01 | 4.37 | **.022** | .12 | 3.38 |
| Time | **<.001** | .37 | 1874.82 | **.031** | .11 | 0.78 | **<.001** | .454 | 22369.47 |
| Time x Relevance | .595 | .01 | < 0.001 | *.096* | .067 | 2.76 | .993 | <.001 | <.001 |
| Time x Group | .977 | <.01 | 0.001 | .285 | .03 | 10.75 | .613 | .006 | <.001 |
| Relevance x Group | .659 | .01 | 0.35 | .651 | .005 | 42.62 | .847 | .001 | 2.35 |
| Group x Relevance x Time | .600 | .01 | 0.007 | .556 | .009 | 209.59 | .434 | .015 | .003 |
| After ~1^st^ SWS period | |  |  |  |  |  |  |  |  |
| Group | .296 | .03 | 2.33 | .244 | .03 | 2.76 | .583 | .01 | 2.94 |
| Relevance | **.006** | .17 | 8.70 | .159 | .048 | 4.08 | *.052* | .089 | 0.62 |
| Relevance x Group | .878 | <.01 | 1.19 | .544 | .009 | 39.68 | .822 | .001 | 6.48 |
| After ~1 week |  |  |  |  |  |  |  |  |  |
| Group | .279 | .03 | 2.17 | .748 | .003 | 3.55 | .299 | .03 | 2.29 |
| Relevance | **.015** | .14 | 3.41 | .262 | .03 | 2.28 | **.040** | .10 | 1.64 |
| Relevance x Group | .517 | .01 | 0.46 | .839 | .001 | 25.27 | .563 | .008 | 3.94 |
| Interference task |  | | | | | | | | |
| Interference learning  after ~1^st^ SWS period | | | |  |  |  |  |  |  |
| Group | .139 | .053 | 1.16 | .219 | .037 | 1.79 | .178 | .044 | 1.49 |
| Relevance | **.021** | .123 | 2.49 | .900 | <.001 | 4.45 | **.018** | .129 | 2.92 |
| Relevance x Group | .905 | <.001 | 1.53 | .900 | <.001 | 27.29 | .864 | .001 | 1.62 |
| Interference recall  after ~1 week | | | |  |  |  |  |  |  |
| Group | .418 | .016 | 3.01 | .865 | .001 | 3.53 | .709 | .004 | 3.31 |
| Relevance | **.030** | .112 | 3.01 | .794 | .002 | 4.34 | .150 | .051 | 1.48 |
| Relevance x Group | .892 | <.001 | 3.31 | .980 | <.001 | 50.78 | 935 | <.001 | 17.17 |

BF: BF_10_ for p < .05, BF_01_ for p > .05; Factor “Group”: cueing/placebo, Factor “Relevance”: relevant/irrelevant, Factor “Time”: after ~1^st^ SWS period/after ~1 week. Grey column highlights p values, significances are indicated in bold, trends are indicated in italic.
